# Endoscopic submucosal dissection-assistance robot: a miniature surgical manipulator for endoscopic submucosal dissection

**DOI:** 10.1055/a-2234-4160

**Published:** 2024-01-23

**Authors:** Yaxian Kuai, Jie Peng, Jing Jin, Aijiu Wu, Derun Kong

**Affiliations:** 1 36639 Key Laboratory of Digestive Diseases of Anhui Province, Department of Gastroenterology, First Affiliated Hospital of Anhui Medical University, Hefei, China; 2 Research and Development Department, Hefei Zhongna Medical Instrument Co. Ltd., Hefei, China

One-handed operation and gravity-induced blind dissection are technical difficulties in endoscopic submucosal dissection (ESD) procedures 1 . In order to reduce the difficulty of the procedure and minimize the risk of perforation and bleeding, we have developed a microsurgical manipulator, the ESD-assistance robot, which provides a “second hand” with auxiliary traction, clamping, and fixation functions during procedures.

The ESD-assistance robot is composed of four main components: an end cap sheath tube, manipulator, servo driver, and command controller ( Fig. 1 ). When in use, the end cap sheath tube is fitted at the front end of the endoscope, and the endoscope is extended to extend the sheath tube. When performing dissection, the micromanipulator is voice controlled to move in forward/backward and clockwise/counterclockwise rotational directions, to bend forward/backward, and to open/close the mouth of the clamp ( Fig. 2 ); auxiliary traction is applied to the pathologic tissue, maintaining the surgical field of view, thereby improving the dissection speed and reducing the difficulty of the procedure.

**Fig. 1:**
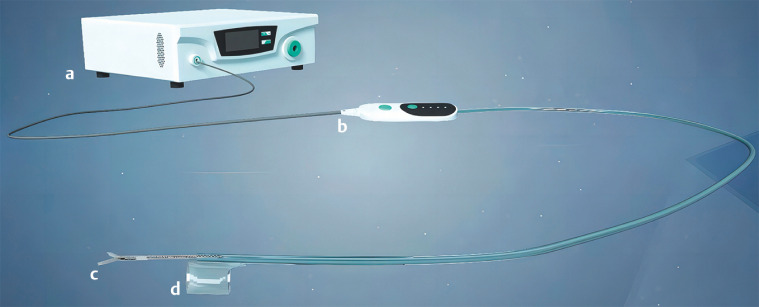
Endoscopic submucosal dissection-assistance robot consists of: **a** a command controller; **b** a servo driver; **c** a manipulator; **d** an end cap sheath tube.

**Fig. 2:**
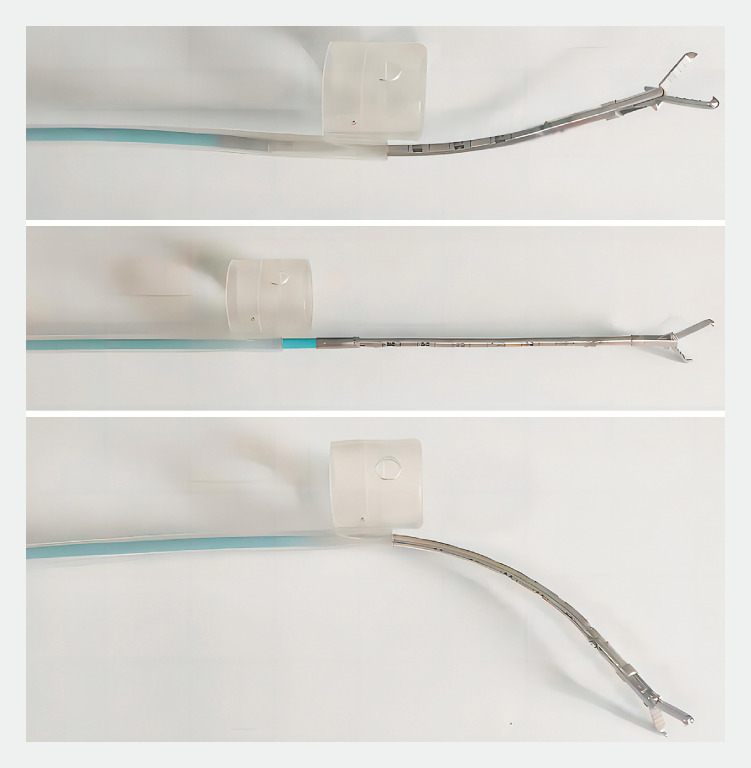
Photographs illustrating the movements of the manipulator.

A 60-year-old man was admitted to hospital with gastric cardia mucosal lesions and underwent diagnostic excision by ESD. During the procedure, traction from the ESD-assistance robot was used to obtain a clear submucosal field of vision, and to facilitate dissection, hemostasis, and submucosal injection ( Fig. 3 ), which together improved surgical efficiency ( Video 1 ).

**Fig. 3:**
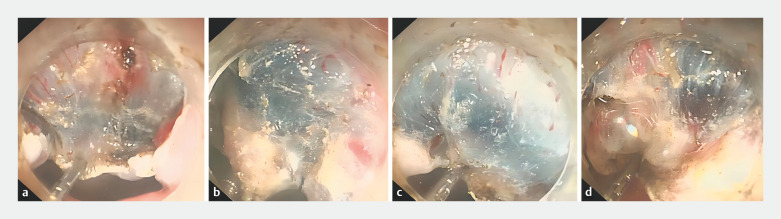
Endoscopic views of the endoscopic submucosal dissection (ESD)-assistance robot being used to assist with the application of traction, and with clamping and fixation.

Application of the endoscopic submucosal dissection (ESD)-assistance robot in gastric ESD.Video 1

Endoscopy_UCTN_Code_TTT_1AO_2AC

## Notes

Endoscopy E-Videos https://eref.thieme.de/e-videos
*E-Videos* is an open access online section of the journal *Endoscopy* , reporting on interesting cases and new techniques in gastroenterological endoscopy. All papers include a high-quality video and are published with a Creative Commons CC-BY license. Endoscopy E-Videos qualify for HINARI discounts and waivers and eligibility is automatically checked during the submission process. We grant 100% waivers to articles whose corresponding authors are based in Group A countries and 50% waivers to those who are based in Group B countries as classified by Research4Life (see: https://www.research4life.org/access/eligibility/ ). This section has its own submission website at https://mc.manuscriptcentral.com/e-videos .
